# The Bi-directional Relationship between Source Characteristics and Message Content

**DOI:** 10.3389/fpsyg.2018.00018

**Published:** 2018-01-30

**Authors:** Peter J. Collins, Ulrike Hahn, Ylva von Gerber, Erik J. Olsson

**Affiliations:** ^1^Reasoning and Argumentation Lab, Department of Psychological Sciences, Birkbeck University of London, London, United Kingdom; ^2^Department of Philosophy, Lund University, Lund, Sweden

**Keywords:** evidence, argument, source reliability, epistemology, Bayesian models

## Abstract

Much of what we believe we know, we know through the testimony of others (Coady, 1992). While there has been long-standing evidence that people are sensitive to the characteristics of the sources of testimony, for example in the context of persuasion, researchers have only recently begun to explore the wider implications of source reliability considerations for the nature of our beliefs. Likewise, much remains to be established concerning what factors influence source reliability. In this paper, we examine, both theoretically and empirically, the implications of using message content as a cue to source reliability. We present a set of experiments examining the relationship between source information and message content in people's responses to simple communications. The results show that people spontaneously revise their beliefs in the reliability of the source on the basis of the expectedness of a source's claim and, conversely, adjust message impact by perceived reliability; hence source reliability and message content have a bi-directional relationship. The implications are discussed for a variety of psychological, philosophical and political issues such as belief polarization and dual-route models of persuasion.

## Introduction

When a doctor recommends a treatment, a patient does not have to conduct a literature review before consenting. The patient can use the doctor's claim and her status as a source to fix the resulting attitude, belief, or action. Like this patient, we learn not just from our own experience but also from other people. How do we treat people as sources? When do we (dis-)trust their claims? These enduring questions arise in classic research on the “wisdom of the crowds” (see, e.g., Galton, 1907), research on judgment and decision making (e.g., Birnbaum et al., 1976; Birnbaum and Stegner, 1979; Birnbaum and Mellers, 1983), and research on persuasion (e.g., Petty and Cacioppo, 1984, 1986; Chaiken et al., 1989). It should thus come as no surprise that research interest in trust and source reliability has continued to grow, with fresh impetus in the study of trust in developmental psychology (for a review, see Mills, 2013), in computer science (for a review, see Artz and Gil, 2007), and in philosophy (e.g., Coady, 1992; Bovens and Hartmann, 2002, 2003). Furthermore, in these contexts, there are not only questions about when we do trust people, but also about whether we do so rationally. How *should* we integrate other people's claims into our own beliefs? These are key questions given that real-world sources are generally less than fully reliable (see, e.g., Bovens and Hartmann, 2002, 2003).

Within psychology, it is the study of persuasion that has treated sources most extensively. Early theories of persuasion centered on a putative dichotomy between the content of persuasive messages and their sources (for a review, see Petty and Briñol, 2008). Hovland and colleagues, for instance, argued that persuasion could arise as a function of either learning a substantive argument or learning simple cues such as the source's characteristics (e.g., Kelman and Hovland, 1953). A dichotomy between message and source became central to the dominant dual-process theories of persuasion, such as the Elaboration Likelihood Model (“ELM;” e.g., Petty et al., 1981; Petty and Cacioppo, 1984, 1986) and the Heuristic-Systematic Model (e.g., Chaiken et al., 1989). They comprise two routes to persuasion: central (focused on arguments; analytical, systematic, high elaboration) and peripheral (focused on general impressions and surface features; heuristic, low elaboration).

Contemporary dual-process theories recognize that message content and sources can interact in subtle ways. The ELM identifies five ways in which sources can induce persuasion (Petty and Briñol, 2008; Briñol and Petty, 2009). (1) Under conditions of low elaboration, when recipients are unmotivated or unable to think about a particular issue, sources can act as simple, heuristic cues. A classic study showed that, when personal relevance was low, persuasiveness was due to source reliability; when personal relevance was high, persuasiveness was due to argument strength, that is, the actual content of the persuasive message (Petty et al., 1981). (2) Under conditions of high elaboration, sources can act as an argument or evidence. When an attractive source testifies to the effectiveness of a beauty product, the source's appearance is visual evidence for the effectiveness of the product (Petty and Briñol, 2008). In other words, source characteristics can, occasionally^1^, have evidential value on the “analytic” route. (3) Sources can affect metacognition. For example, when source information comes after an argument, credible sources can increase people's confidence in their thoughts (Briñol et al., 2004). (4) Sources can bias thinking. For example, source expertise can affect the direction of thoughts, so long as the message is ambiguous and the task is important (Chaiken and Maheswaran, 1994). (5) Sources can affect the extent of thinking. For example, when there are multiple sources for a claim, people tend to think longer, magnifying differences attributable to argument strengths: strong arguments become more persuasive; weak arguments, less persuasive (Harkins and Petty, 1981). In other words, (4) and (5) allow source information to affect analytic processing in ways that go beyond evidential value, by moderating the direction and amount of analytic thinking that takes place.

Hence, the contemporary ELM provides a subtler account of sources than earlier perspectives, no longer confining source information to the peripheral route. But there remain challenges. Sometimes, for instance, an intuitively good and complex argument depends principally (or even solely) on information about its source, as when arguments for anthropogenic climate change are based on the beliefs of climate scientists (see Hahn et al., 2015). Other times both content and source information seem relevant. In such cases, how should we combine the information; how separable are the two types? Where the ELM has addressed this question, it has suggested that argument and source provide *additive* cues. If source characteristics are deemed “informative and relevant when scrutinized” (such as in the case of the attractiveneness of the person advertising beauty products) they provide an independent potential argument supporting the advocacy of the message, which “adds to the impact of the other information” within the analytic route (Petty and Wegener, 1998, p. 52). This position is explicitly contrasted with that of Chaiken's Heuristic Systematic Model (HSM) where both routes may interact in processing argument content and source (see also, Maheswaran and Chaiken, 1991; Ratneshwar and Chaiken, 1991).

The persuasion literature echoes a large and venerable prescriptive literature on argumentation. In this literature, arguments are supposed to speak for themselves. Where arguments rely on source information, they are deemed fallacious, as, for example, with *ad hominem* arguments, which attack the credibility of the source, or appeals to authority, which are based on the source's credibility. Such arguments feature prominently in traditional catalogs of fallacies (e.g., Woods et al., 2004) and textbooks on critical thinking (e.g., Bowell and Kemp, 2002; Hughes et al., 2010; Rainbolt and Dwyer, 2012). However, even this tradition increasingly holds that such arguments are sometimes reasonable, and focuses on distinguishing fallacious and non-fallacious forms (e.g., Walton, 1998; van Eemeren et al., 2009). But recent work goes further still, and argues that source characteristics have evidential value in a broad range of circumstances (e.g., Hahn et al., 2009, 2013; Oaksford and Hahn, 2013). This work adopts a normative, Bayesian perspective which mandates sensitivity to source characteristics in many argument evaluation contexts. This perspective is echoed in Bayesian treatments of testimony in the context of developmental psychology (Shafto et al., 2012), legal testimony (e.g., Schum, 1981; Friedman, 1987; Lagnado et al., 2013), or the value of the level of consensus among climate scientists (Hahn et al., 2015).

This Bayesian approach to argumentation is an instance of a more general approach to cognition, where optimal models are developed and compared with data from participants. This approach has been applied, for instance, to perception (e.g., Geisler, 1987), categorization (e.g., Anderson, 1991), or syllogistic reasoning (Chater and Oaksford, 1999). These models presuppose Bayesianism on the grounds that, under certain conditions, Bayesian reasoning is demonstrably optimal (Rosenkrantz, 1992; Leitgeb and Pettigrew, 2010a,b; for discussion, see Hahn, 2014). If human behavior approximates the model, then the optimal model provides a functional explanation of why human behavior is the way it is. However, such models are also useful where deviations arise as they can guide exploration of constraints that underlie the shortfall between actual and optimal behavior (see, e.g., Geisler, 1987; Anderson, 1990; Howes et al., 2009).

In argumentation, this approach has given rise to hypotheses that have prompted experimental research on the influence of source characteristics in the context of argument (see e.g., Hahn et al., 2009; Harris et al., 2012, 2013, 2015). Many of these hypotheses originate in Bayesian approaches to testimony, that is, belief updating in response to the saying, uttering, asserting of a claim by a source of partial reliability (e.g., Bovens and Hartmann, 2002, 2003; Olsson, 2005). Here, normative Bayesian models prescribe that message content and source reliability should be considered together to avoid the mis-calibration of beliefs. Such models often yield surprising, counter-intuitive results. For instance, diverse evidence (e.g., evidence from independent sources) is not always more compelling (Bovens and Hartmann, 2003), and pieces of testimonial evidence that “fit together” or cohere are not necessarily more likely to be true (see e.g., Olsson, 2005; for an empirical investigation of coherence, see also Harris and Hahn, 2009). Finally, where multiple pieces of testimonial evidence are concerned, there will, normatively, be subtle, complex, interactions between the reliability of the individual witnesses, and how informationally independent they are from one another (see e.g., Hahn et al., 2015 for an overview).

Initial experimental evidence suggests that people conform, to some extent, to Bayesian norms. Even when participants evaluate arguments in fictitious scenarios that should promote conditions of low personal involvement from the perspective of the ELM, they are, in fact, sensitive to *both* message content and message source, and their behavior shows interactions between content and source reliability (Hahn et al., 2009). Such behavior is, at least qualitatively, consistent with Bayesian norms. Specifically, interactions arise from the multiplicative nature of Bayes' rule (the central rule for belief revision in Bayesian models).

In this paper, we consider two specific models which prescribe consideration of messages and sources together. The models apply under conditions of uncertainty, and tell us how to update our beliefs: that we should follow Bayes' rule. Of course, in the real world, sources are generally fallible, hence only partially reliable, but their precise degree of reliability is also not known. The Bayesian approach does not tell us how to judge the initial reliability of our sources. Literature on lie detection, for example in forensic contexts, has considered individual features that might be informative about whether or not a source is telling the truth, ranging from personality characteristics to mannerism or behaviors, such as voice characteristics, gestures, or eye movements (see, e.g., DePaulo et al., 2003; Vrij et al., 2010). But intuitively relevant, too, is the actual content of what someone says. This is obviously the case where it is known that what someone has claimed is actually false. Whether this was based on an intentional lie or merely an error, it should clearly affect our views about the reliability of the individual concerned. However, philosophers concerned with testimony have also taken the view that we might already consider relevant to judgments of reliability any statements that strike us as *implausible*, even though we are willing to allow the possibility that they are, in fact, correct.

From the literature on formal epistemology, two related Bayesian models have embodied this intuition: the model of Bovens and Hartmann (2003) and that of Olsson and Angere (reported in, e.g., Olsson, 2011; Olsson and Vallinder, 2013). These models share a fundamental assumption: that message content and source reliability interact bi-directionally. On the one hand, the reliability of the source moderates the evidential impact of the message content. On the other hand, message content provides evidence about the reliability of the source. Effectively, hearing someone say something implausible or unexpected (e.g., “the Earth is flat”) leads to a reduction in the probability (subjective degree of belief) that they are reliable. Both are Bayesian models in that they use Bayes' rule to update beliefs both about what it is a source is asserting and about the source's reliability. However, they differ in detail, particularly with respect to what it means for a source to be “unreliable.”

### Two bayesian models of source reliability

**Figure 1:**
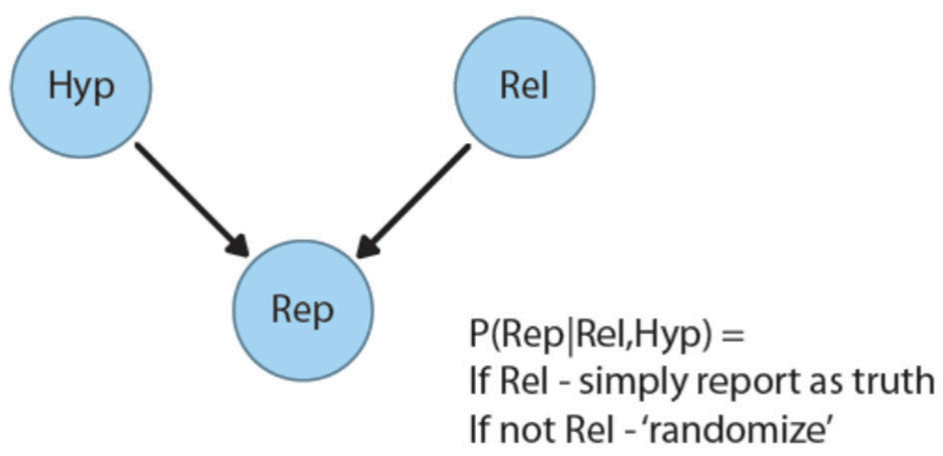
Bayesian belief network of testimony from Bovens and Hartmann (2003).

The Bovens and Hartmann's model is illustrated by the simple Bayesian Belief Network in Figure 1 below^2^. In this model, A source makes a report (represented in the network by the binary variable Rep), “X is true (false):” the state of this binary report variable depends on both the underlying state of the world (represented by the node HYP, for “hypothesis”) *and* the reliability of the source (represented by the binary variable “Rel”). If the source is reliable, it is simply assumed to report the true state of affairs. If the source is unreliable, however, its report has no systematic connection with the world—it is as though a coin is flipped to determine whether to assert the truth or the falsity of what is being reported (though different degrees of bias toward positive or negative reports can be modeled as well; see Bovens and Hartmann, 2003, for details).

On hearing a report, the recipient revises both her belief in the hypothesis and her belief in the reliability of the source. After an unexpected message [*P(HYP)* < 0.5], reliability *P(REL)* will be revised downward, as in the flat Earth example above. After a plausible, expected, message [*P(HYP)* > 0.5], belief in the source's reliability will go up. Within the psychological literature, consequences of this simple model have been explored, for example, in Jarvstad and Hahn (2011).

Olsson and Angere's model differs in two principal ways. Firstly, source reliability is represented not by a binary variable, but by a distribution over possible reliability profiles, updated via Bayesian inference. Secondly, unreliability does not lead to randomization. In Bovens' and Hartmann's model, “unreliable” means uncorrelated with the truth: unreliability bottoms out at *P* = 0.5. In Olsson and Angere's model, unreliability bottoms out at *P* = 0: a maximally unreliable source is negatively correlated with the truth. We will call this “*anti-reliability*.” Here, the response is to take the report as evidence of *the opposite* of what is being asserted. For example, a used-car dealer saying that one vehicle is better than the other is taken as evidence of the opposite. Unreliability in the sense of random responding is simply one of many possible reliability profiles along the continuum from “completely reliable” to “anti-reliable,” and a given source can, in principle, adopt any point along the way.

Together the models raise empirical questions about what people do. Do people use message content to revise their beliefs about a source, and, in particular, do they do so even in a minimal context where there is no other information? If they do so, do they use message content to revise beliefs about reliability both upwards and downwards? And, finally, under what circumstances, if any, are they willing to consider sources to be anti-reliable?

These questions are of theoretical interest: they are important not only to projects within epistemology aimed at understanding the concept of “knowledge,” but also to the models of the impact of source characteristics on rational argument that have been built around them (e.g., Harris et al., 2012, 2015). And these, in turn, as discussed above, are of interest to anyone concerned with persuasion and the role of source characteristics in the psychological processing of persuasive messages. However, the question of whether there is a bi-directional relationship between message content and perceived source reliability is also of wider societal importance. Perceived anti-reliability, for instance, may help to explain belief polarization, whereby collectives might find themselves split into groups of ever more extreme, diametrically opposing views (for a discussion of belief polarization in US politics, see Mann and Ornstein, 2012). Polarization may ensue rapidly once opponents, say, Republicans and Democrats take evidence offered by the other group to, anti-reliably, be evidence to the contrary. Indeed, simulations with societies of artificial agents based on the Olsson and Angere model typically develop this kind of belief polarization within the group (Olsson and Vallinder, 2013; see also Hahn and Harris, 2014). It thus matters greatly, from a practical perspective, whether anti-reliability requires special kinds of evidence, or whether it might arise simply from the fact that the content of communications seems unexpected.

This paper presents a series of experiments that explore whether message content influences perceived source reliability and vice versa. Experiment 1a examined the extent to which participants changed their beliefs in response to claims presented by more or less reliable (expert, trustworthy) sources. Experiment 1b examined whether participants revised their perceptions of source reliability after expected and unexpected claims (low/high prior probability). Experiment 2a and 2b provide a replication. Experiment 3, finally, employed a different method, which avoided any overt reference to source reliability, to examine further the extent to which participants spontaneously use message content to revise beliefs about message source.

The main hypotheses, following on from Bovens and Hartmann's (BH) and Olsson and Angere's (OA) basic models, were as follows:

Experiments 1a and 2a examined the effects of reliability on beliefs. Specifically, they tested the prediction that reliable sources should increase belief in a claim. This prediction is common to both the BH and OA models. Only the OA model, however, predicts that unreliable sources could *decrease* belief in a claim, that is, unreliable sources may be viewed as “anti-reliable” prompting belief change in the opposite direction of what they assert. The alternative prediction of the BH model is that maximally unreliable sources are simply viewed as uninformative, so that beliefs do not change in response to messages from them.

Experiment 1b and 2b examined the converse relationship, that is, the effects of claims on perceived reliability. For both models (BH and OA) expected claims should increase source reliability and unexpected claims should decrease source reliability.

Experiment 3, finally, tested for implicit effects of message content on source reliability by examining the impact of a message on beliefs as a function of a preceding message by the same source. On both accounts (BH and OA) a second claim should be more convincing following an expected claim. Only the OA account additionally allows for possible anti-reliability such that an initial unexpected claim could change the valence of a second claim.

## Experiments 1A and B

The aim of Experiments 1a and 1b was to examine the (putatively) tight connection between source and content with a single set of materials involving a factorial combination of expected/unexpected claim and reliable/unreliable source. Participants were either asked to evaluate the claim (Exp. 1a) or the source (Exp. 1b). These materials could be used to examine either the effect of reliability on message convincingness or the effect of message convincingness on source reliability, depending on the claim (Exp. 1a) or the source (Exp. 1b).

### Methods

Both experiments followed a 2 × 2 between-subjects design with the following factors: Claim Expectedness (Expected, Unexpected) and Source Reliability (High, Low).

#### Experiment 1a: belief in a claim

##### Participants

Ninety-nine people (45 women; average 38.63) gave informed consent and completed online surveys hosted on a US-hosted website for academic research (http://psych.hanover.edu/research/exponnet.html), with participants largely recruited through university e-mail lists at Lund University, Sweden.

##### Materials and procedure

Participants read brief texts about six topics. Each text took the following form. Participants first read a claim and rated its convincingness by responding to the question “How convincing is the claim?” on a Likert-style scale from 0 (not at all convincing) to 10 (completely convincing). For example,

“One of the best remedies against a severe cough is valium.”

Participants were then presented with a source making this claim:

Now imagine that Michael, who is a clinical nurse specialist, told you the following: “One of the best remedies against a severe cough is valium.”

Following this, participants re-rated the convincingness of the claim on the same Likert-scale.

Other participants saw corresponding versions of the same scenario that differed in the reliability of the source and/or the expectedness of the claim. For the present example, the expected claim was “One of the best remedies against a severe cough is lots to drink, hot or cold,” and the unexpected claim was “One of the best remedies against severe cough is valium.” The reliable source was “Michael, who is a clinical nurse specialist,” whereas an unreliable source was “Michael, who is a drug addict.”

Each participant saw a set of six such scenarios drawn from one of the four conditions; half of the participants saw the same sets with the respective orders reversed to control for order effects. The initial ratings act as a manipulation check, with reliable differences in expectedness in the anticipated directions. These data are summarized in Table 1, Appendix 2 in Supplementary Material.

For the full set of materials, see the Appendix in Supplementary Material^3^.

#### Experiment 1b: perceived reliability

##### Participants

One hundred and thirty-one people (45 women; average age 39.83) gave informed consent and completed online surveys hosted on a US-hosted website for academic research (http://psych.hanover.edu/research/exponnet.html), with participants largely recruited through university e-mail lists at Lund University, Sweden.

##### Materials and procedure

Participants read texts on the same six topics as in Experiment 1a. The only difference concerned the dependent variables. Instead of providing an initial judgment on the convincingness of the claim, the participants first read about the source and rated its reliability by responding to the question “How reliable do you think [source name] is?” on a Likert-style scale from 0 (not at all reliable) to 10 (completely reliable). Next, participants read the same source information again, but this time together with a claim. For example, having read that “Michael is a drug addict,” some participants read the following:

Now imagine that Michael told you the following: “One of the best remedies against a severe cough is valium.”

Participants re-rated source reliability on the same Likert scale. No definition of “reliability” was provided. As in 1a, each participant saw a script with six texts, with two orders of presentation to control for order effects. For the full set of materials, see the Appendix in Supplementary Material. Once again, the initial ratings act as a manipulation check, with reliable differences in reliability in the anticipated directions. These data are summarized in Table 1, Appendix 2 in Supplementary Material.

### Results

We chose to run Bayesian analyses for all experiments reported in this paper: specifically, robust Bayesian parameter estimation. The analyses are, in effect, Bayesian equivalents of classical one-sample *t*-tests (for Experiments 1 and 2) and independent-sample *t*-tests (for Experiment 3). The Bayesian analyses are useful because they provide richer information than the classical tests—posterior distributions over parameter values—and are not dependent either on assumptions about the data (e.g., normality) or on sampling intentions (Kruschke, 2013). The Bayesian analyses are also invaluable when testing models, because the analyses can lead to both rejection and acceptance of the null hypothesis (Kruschke, 2013).

For Experiments 1a and b, we calculated change scores by subtracting the initial item rating from the final item rating. We then averaged across items (scenarios) to create a mean change score for each participant. We then entered the data into analyses following the guidelines in Kruschke (2013). These analyses do not assume that the data are normally distributed, but instead describe the data with a *t*-distribution, which allows heavy tails. *T*-distributions have three parameters: the mean, *μ*; standard deviation, *σ*; and normality, *ν*. Where the value of the normality parameter is large (ca. 100), the distribution is nearly normal; where it small, the distribution is heavy tailed (Kruschke, 2013).

The one-group analyses for Experiments 1a and b estimate the most credible parameter values, given the data, for the following model:

$$\begin{array}{l} \mathrm{Pr}\left(\mu ,\sigma ,\nu |D\right)=\frac{\text{Pr}\left(D|\mu ,\sigma ,\nu \right)\;\times \;Pr\left(\mu ,\sigma ,\nu \right)\;}{\text{Pr}\left(D\right)} \end{array}$$

The denominator is approximated using Markov Chain Monte Carlo (MCMC) methods, which simulate thousands of combinations of parameter values (for more technical details, see Kruschke, 2013). We ran the analyses in R (R Core Team, 2015) and JAGS using the packages BEST (Meredith and Kruschke, 2013) and rjags (Plummer, 2003). We used the default values of the BEST programs (see Kruschke, 2013). By default, the MCMC chain has 100,000 steps, with no thinning to correct for autocorrelation. The default priors are uninformative. Since this is, to the best of our knowledge, the first study to these content/source predictions, uninformative priors are justified. The prior for *μ* is centered on the mean of the data, the spread being determined by the precision equivalent to 100 times the standard deviation; for *σ* it is a broad uniform distribution from 1/1,000 to 1,000 times the standard deviation of the data; for *ν* it is an exponential distribution giving roughly equal credibility to nearly normal and heavy-tailed distributions (for further details, see Kruschke, 2013).

The remainder of this section reprises the predictions, and reports the corresponding posteriors for the parameters. To decide whether the parameter estimates (dis-)confirm the predictions, we need two further concepts: the highest density interval (HDI) and the regional of practical equivalence (ROPE). The HDI spans the most credible (highest probability) values of the posterior distribution: for instance, a 95% HDI, which we will use throughout, covers 95% of the distribution, and the values within it have a total probability of 0.95 (Kruschke, 2013, 2015). When assessing predictions, we can ask whether the 95% HDI includes a specific point value: for example, for the null hypothesis, zero. In reality, requiring a point value may be too stringent. In such cases, a ROPE can prove helpful: values within this region are considered practically equivalent to the comparison value. Kruschke (2015) recommends that, in the absence of clear guidelines in the field, researchers establish a ROPE around the comparison value, from −0.1 to 0.1. Below, we will apply the ROPE to effect sizes, so that the relevant comparison value will be zero with a ROPE from −0.1 to +0.1. We will base our evaluations of the experimental predictions on these effect sizes and corresponding ROPEs. If the 95% HDI falls entirely outside of the ROPE, there is a clear effect; if it falls entirely within the ROPE, there is a null effect. In this case, the 95% HDI for effect size falls outside this conventional ROPE. Where there is overlap, the data do not allow a clear decision for the specific HDI and ROPE. It may, nevertheless, be informative to consider how much overlap there is, as this will give some indication of weaker conclusions.

#### Experiment 1a: belief in claim

(1) Reliable sources should increase belief in a claim
(2) (i) Unreliable sources should decrease belief in a claim. OR
      (ii) Unreliable sources should not affect belief in a claim.

**Figure 2:**
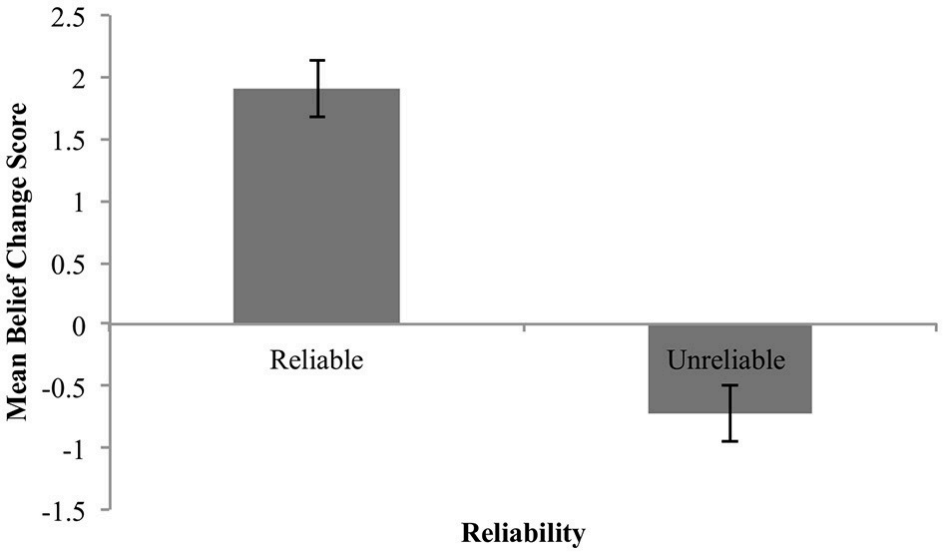
Mean belief change for reliable and unreliable sources. Error bars are standard error.

Figure 2 shows the mean belief change for the claim (collapsed across claim expectedness).

These means show the predicted increase in belief in the claim in response to testimonial evidence from a reliable source, and a *decrease* in response to the same evidence when coming from an unreliable source. In other words, the data are suggestive of anti-reliability (2i).

We statistically evaluated these findings with two one-group analyses with a comparison value of 0, analogous to classical one-sample *t*-tests^4^.

##### Reliable sources

**Figure 3:**
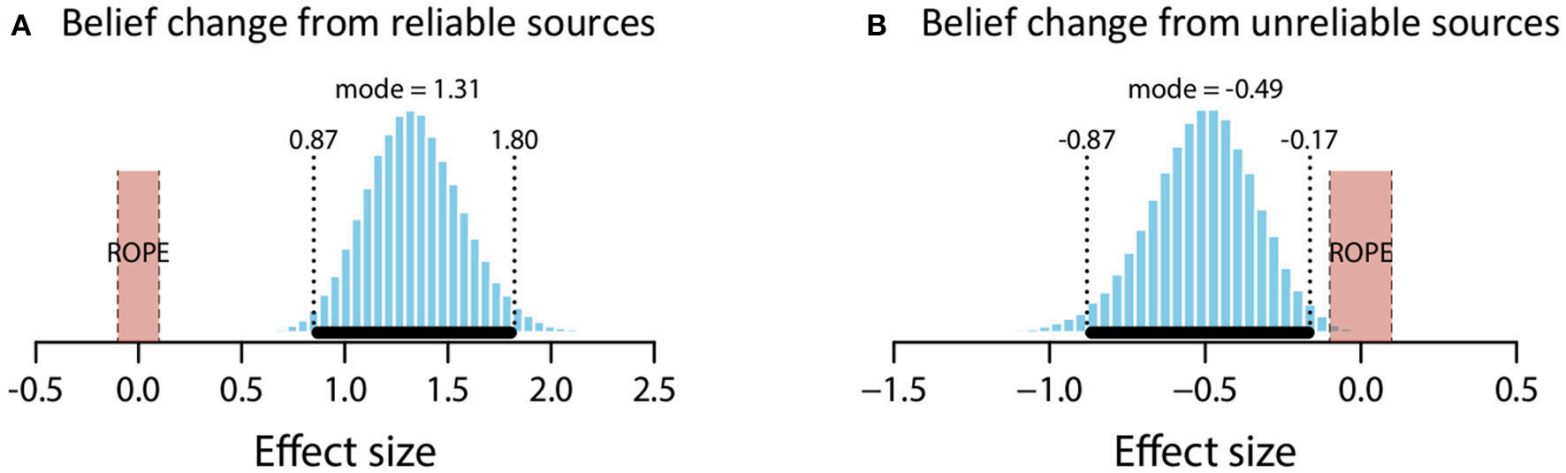
Posterior distributions of effect size for belief change from reliable sources **(A)** and unreliable sources **(B)**. ROPE from −0.1 to 0.1. Black bar represents 95% HDI.

The mean estimate for *μ* was 1.84 (95% HDI [1.37, 2.3]). The modal estimate for *σ* 1.39 (95% HDI [1.01, 1.81]). The modal estimate for log_10_(*ν*) was 1.37 (95% HDI [0.42, 2,04]). Lastly, the modal estimate for effect size—(*μ*-0)/*σ*–was 1.31 (95% HDI [0.87, 1.8]), which falls outside the conventional ROPE. Figure 3A shows the posterior distribution for effect size and the ROPE. This analysis, then, shows that reliable sources credibly increased belief in a claim.

##### Unreliable sources

The mean estimate for *μ* was −0.72 (95% HDI [−1.15, −0.29]). The modal estimate for *σ* was 1.46 (95% HDI [0.97, 1.88]). The modal estimate for *ν* was 1.36 (95% HDI [0.37, 1.99]). Lastly, the modal estimate for effect size was −0.49 (95% HDI [−0.87, −0.17]), which falls outside the conventional ROPE. Figure 3B shows the posterior distribution for effect size and the ROPE. Thus, this analysis shows that unreliable sources credibly decreased belief in a claim.

##### Summary

These data therefore support both predictions (1) and (2)(i): reliable sources increased belief in a claim; unreliable sources decreased belief in a claim. The data offer support, then, for source anti-reliability.

#### Experiment 1b: perceived reliability

(3) Expected claims should increase source reliability
(4) Unexpected claims should decrease source reliability.

**Figure 4:**
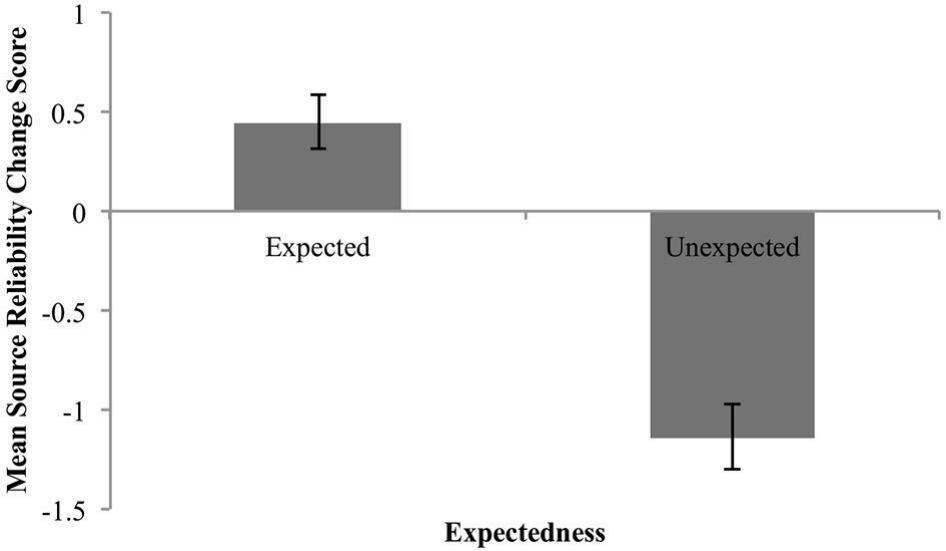
Mean change in perceived reliability for expected and unexpected claims. Error bars are standard error.

The mean change in the perceived reliability of the source as a function of claim expectedness or unexpectedness is shown in Figure 4 below. These means are in keeping with (3) and (4): expected claims led to increases in source reliability, unexpected claims to decreases.

We again statistically evaluated the predictions with two one-group analyses with a comparison value of 0.

##### Expected claims

**Figure 5:**
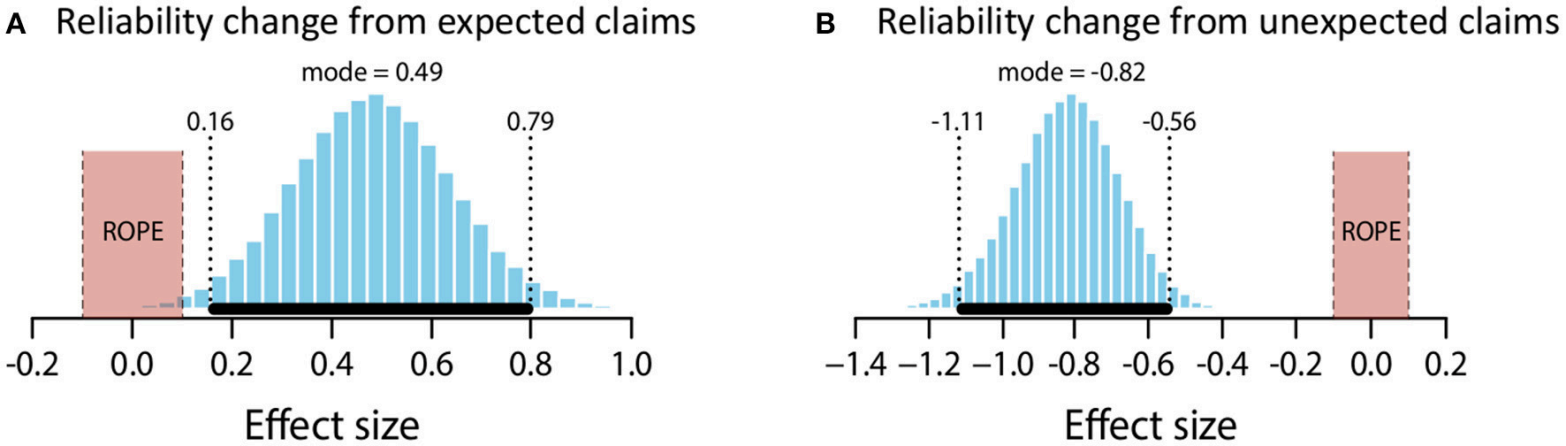
Posterior distributions of effect size for change in perceived reliability from expected claims **(A)** and unexpected claims **(B)**. ROPE from −0.1 to 0.1. Black bar represents 95% HDI.

The mean estimate for *μ* was 0.45 (95% HDI [0.18, 0.74]). The modal estimate for *σ* was .93 (95% HDI [0.75, 1.18]). The modal estimate for log_10_(*ν*) was 1.53. The modal estimate for effect size was 0.49 (95% HDI [0.15, 0.79]), which falls outside the conventional ROPE. Figure 5A shows the posterior distribution for effect size and the ROPE. Thus, this analysis shows that expected claims credibly increased source reliability.

##### Unexpected claims

The mean estimate for *μ* was −1.12 (95% HDI [−1.43, −0.8]). The modal estimate for *σ* was 1.37 (95% HDI [1.09, 1.65]). The modal estimate for log_10_(*ν*) was 1.16 (95% HDI [0.59, 1.96]). The modal estimate for effect size was −0.82 (95% HDI [−1.11, −0.56]), which falls outside the conventional ROPE. Figure 5B shows the posterior distribution for effect size and the ROPE. Thus, this analysis shows that unexpected claims credibly decreased source reliability.

##### Summary

These data therefore support predictions (3) and (4). Expected claims increased source reliability; unexpected claims decreased source reliability.

### Discussion

This study is, to the best of our knowledge, the first to test and find support for the view that there is a two-way relationship between claims and sources. Not only do sources affect people's response to claims; claims affect people's judgments of a source's reliability.

These data also serve to distinguish between alternative models of source reliability. As we have seen, these models principally differ with respect to unreliable sources. In Bovens and Hartmann (2003) an unreliable source is taken to be uninformative with respect to the truth of a claim, so that reports from an unreliable source cease to have any impact on an agent's beliefs. Olsson and Angere (reported, e.g., in Olsson, 2011), in contrast, go further and allow source anti-reliability: fully unreliable sources should make people actively disbelieve the claim. Our results suggest that, at least in some circumstances, people are happy to consider sources anti-reliable, even in minimal contexts such as the ones we studied.

## Experiments 2a and b

The novelty of the findings argues for replication. Experiments 2a and b sought to replicate the effects using a different sample. The data from Exp. 1 were collected via a university-hosted website for online experimental studies, with a sample consisting largely of self-selecting, interested volunteers from Lund University students and staff. Experiments 2a and b were posted on Amazon Mechanical Turk. Although samples on Mechanical Turk are also not representative of the general population, they are considered more diverse than college samples (Paolacci and Chandler, 2014), and, most importantly, are likely to be different in composition than the sample of Exp. 1a and b. This offers a useful further test of the effects.

### Methods

The experiments followed the same design as Experiments 1a and b: a 2x2 between-subjects design with the following factors: Claim Expectedness (Expected, Unexpected) and Source Reliability (High, Low). Experiment 2a and 2b used the same materials and procedure as Experiment 1a/b. Two minor changes were made to the materials to adapt them for a predominantly US audience. Firstly, for the Stockholm item, temperatures were given in both Centigrade and Fahrenheit. Secondly, for the nightclub item, US locations were given: Manhattan as an expected location for a prestigious nightclub, and Des Moines, Iowa, as an unexpected location. The Range Rover item was also removed because, in Experiments 1a and b, participants' prior beliefs showed that the intended expectedness manipulation had not worked. For the exact materials see the Appendix in Supplementary Material. Once again, the initial ratings act as a manipulation check, with reliable differences in expectedness and reliability in the anticipated directions. These data are summarized in Table 1, Appendix 2 in Supplementary Material.

#### Experiment 2a

##### Participants

Seventy-nine people (27 women; average age 33.38) gave informed consent and completed online surveys posted on Amazon's Mechanical Turk as a small job (HIT). The HIT was posted by an intermediary, MTurk Data. Participants were rewarded with a small fee equivalent to $0.20 per minute, calculated to exceed the rate of the US minimum wage. To maximize engagement and maximize the number of native English speakers, high qualifications were posted. To complete the task, participants needed to be resident in the US, Canada or UK, have a 99% approval rating for their previous HITs, and to have completed 10,000 approved HITs. One participant's data (not included in the above count) was excluded because that participant reported a first language other than English.

#### Experiment 2b

##### Participants

Seventy-nine people (31 women; average 35.09) gave informed consent and completed online surveys posted on Amazon's Mechanical Turk as a small job (HIT). Participants were recruited and rewarded in the same way as Experiment 2a. In addition, a qualification guaranteed that people could not participate if they had previously completed Experiment 2a. As above, one participant's data (not included in the above count) was excluded because that participant reported a first language other than English.

### Results

We analyzed the data using the same method, model and programs as Experiments 1a and b. Recall that the method estimates the parameter values for the mean (*μ*), standard deviation (*σ*) and normality (*ν*).

#### Experiment 2a

(1) Reliable sources should increase belief in a claim.
(2) (i) Unreliable sources should decrease belief in a claim. OR
          (ii) Unreliable sources should not affect belief in a claim.

**Figure 6:**
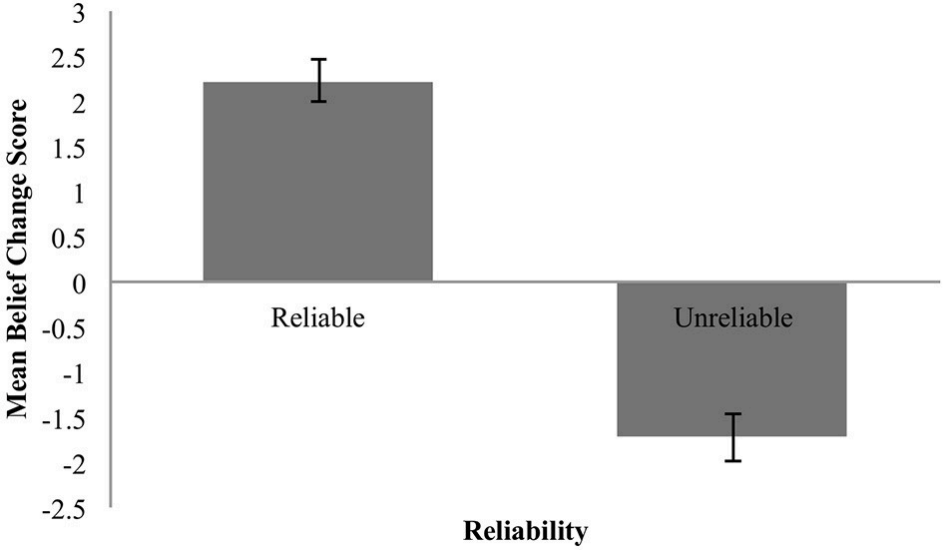
Mean belief change for reliable and unreliable sources. Error bars are standard error.

Figure 6 shows the descriptive data for Experiments 2a. Qualitatively, the same patterns are observed as in Exp. 1 a.

##### Reliable sources

**Figure 7:**
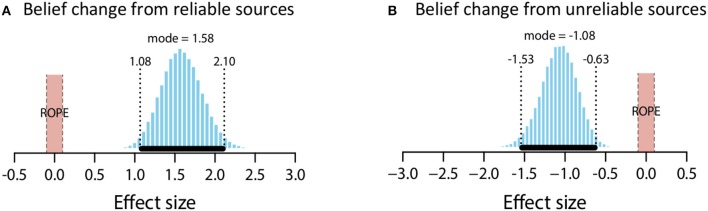
Posterior distributions of effect size for belief change from reliable sources **(A)** and unreliable sources **(B)**. ROPE from −0.1 to 0.1. Black bar represents 95% HDI.

The mean estimate for *μ* was 2.23 (95% HDI [1.76, 2.7]). The modal estimate for *σ* 1.39 (95% HDI [1.09,1.79]). The modal estimate for log_10_(*ν*) was 1.49 (95% HDI [0.75, 2.1]). Lastly, the modal estimate for effect size—(*μ*-0)/*σ*–was 1.58 (95% HDI [1.08, 2.1]), which falls outside the conventional ROPE. Figure 7A shows the posterior distribution for effect size and the ROPE. This analysis, then, shows that reliable sources credibly increased belief in a claim.

##### Unreliable sources

The mean estimate for *μ* was −1.73 (95% HDI [−2.28, −1.18]). The modal estimate for *σ* was 1.58 (95% HDI [1.21, 2.11]). The modal estimate for *ν* was 1.48 (95% HDI [0.6, 2.07]). Lastly, the modal estimate for effect size was −1.08 (95% HDI [−1.53, −6.3]), which falls outside the conventional ROPE. Figure 7B shows the posterior distribution of effect size and the ROPE. This analysis shows that unreliable sources credibly decreased belief in a claim.

##### Summary

These data therefore support both predictions (1) and (2): reliable sources increased belief in a claim; unreliable sources decreased belief in a claim. The data replicate the effects in Experiment 1a, providing further support for source anti-reliability and the Olsson and Angere model.

#### Experiment 2b: perceived reliability

(3) Expected claims should increase source reliability
(4) Unexpected claims should decrease source reliability.

**Figure 8:**
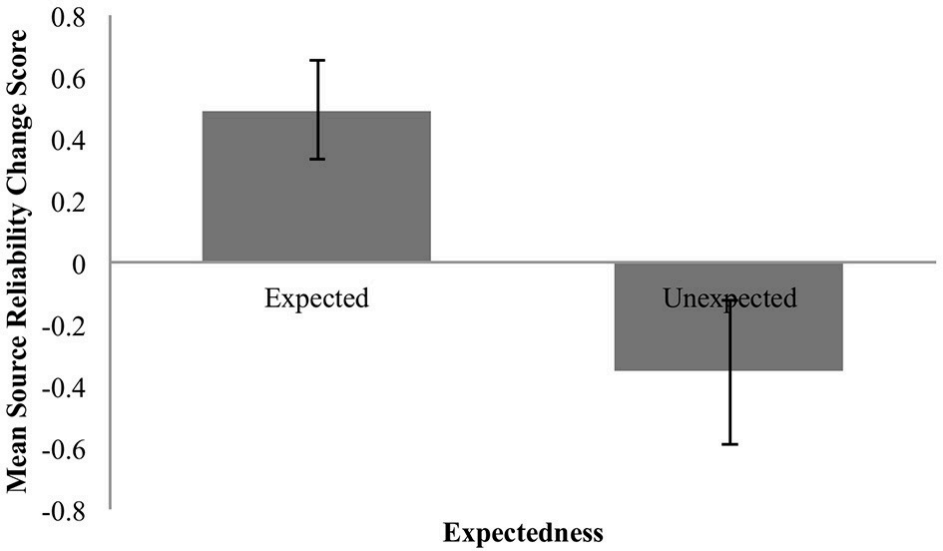
Mean change in perceived reliability for expected and unexpected claims. Error bars are standard error.

Again, the descriptive data (Figure 8) qualitatively match the earlier findings.

##### Expected claims

**Figure 9:**
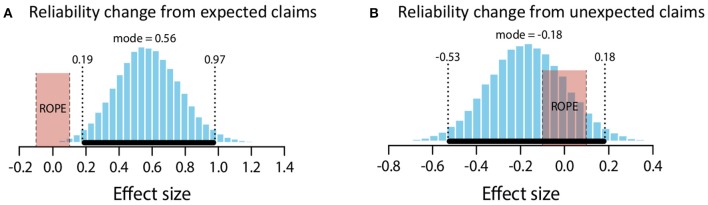
Posterior distributions of effect size for change in perceived reliability from expected claims **(A)** and unexpected claims **(B)**. ROPE from −0.1 to 0.1. Black bar represents 95% HDI.

The mean estimate for *μ* was 0.51 (95% HDI [0.2, 0.83]). The modal estimate for *σ* was 0.89 (95% HDI [0.66, 1.19]). The modal estimate for log_10_(*ν*) was 1.51. The modal estimate for effect size was 0.56 (95% HDI [0.19, 97]), which falls outside the conventional ROPE. Figure 9A shows the posterior distribution of effect size and the ROPE. Thus, this analysis shows that expected claims credibly increased source reliability.

##### Unexpected claims

The mean estimate for *μ* was −0.2 (95% HDI [−0.64, 0.22]). The modal estimate for *σ* was 1.16 (95% HDI [0.65, 1.7]). The modal estimate for log_10_(*ν*) was 0.52 (95% HDI [0.12, 1.77]). The modal estimate for effect size was −0.18 (95% HDI [−0.53, 0.18]). Although this modal estimate is for a small effect, the 95% HDI includes an effect size of 0. Indeed, the 95% includes the entire ROPE (−0.1 to 0.1). It is therefore not possible to reject the null hypothesis. But since the 95% also includes effect sizes outside of the ROPE, it is also not possible to confirm the null hypothesis. Figure 9B shows the posterior distribution and the ROPE. Accordingly, these data do not allow us to make a decision on the effect of unexpected claims.

##### Summary

These data support prediction (3) but (in contrast to Exp. 1b) do not allow a decision on prediction (4). Expected claims increased source reliability; but there was no statistical evidence for (or against) unexpected claims decreasing reliability.

### Discussion

Experiments 2a and 2b broadly support the findings of Experiments 1a and 1b. Participants used source reliability when assessing claim strength, and can consider sources to be anti-reliable or negatively correlated with the truth. Participants also used message content to form impressions of source reliability. The data are also more consistent with Olsson and Angere's normative model of source reliability than with Bovens and Hartmann's.

On one point, Experiments 1b and 2b differ slightly. In Experiment 1b, participants responded to unexpected claims by revising their perception of source reliability downwards. In Experiment 2b, although the estimate of effect size suggested a downgrading of source reliability, the data did not allow a decision between that hypothesis and the null hypothesis.

The experiments thus far have tested participants' responses to single claims. Participants readily revised their beliefs and perceptions of source reliability. For this mechanism to play a major role in belief revision, however, these beliefs and perceptions should hold across multiple interactions. The next experiment explores this possibility.

## Experiment 3

Experiment 3 built on the materials of the previous experiments but moved to a new experimental paradigm. In particular, we wanted to examine further the extent to which people spontaneously use message content to revise their beliefs about a source. We thus used an experimental paradigm that avoided all mention of source reliability. To this end, the task involved multiple claims by the same source. We sought to test for effects of message content on perceived reliability by manipulating the expectedness/unexpectedness of the initial claim and testing for subsequent differences on a second claim, which was the same claim in all conditions. Any systematic differences on this second claim reflect spontaneous and implicit revision of perceived reliability as the scenario described in the text unfolds. There was no probe of either reliability or convincingness for the first claim, nor did the experimental procedure pause on it in any way. In effect, Exp. 3 combines the questions addressed separately in the preceding experiments—namely whether source reliability matters and whether claim expectedness affects reliability—into a single study. As any potential effects of message expectedness on reliability are entirely implicit, the study allows insight into the extent to which people naturally revise their opinions about the reliability of sources on the basis of message content and factor these in to the processing of subsequent messages by those sources.

To accommodate multiple claims, we changed the structure of the task. Participants now read items such as the following:

Imagine you hear Michael, who is a clinical nurse specialist, telling someone “One of the best remedies for a severe cough is valium.” Later, Michael tells you the following: “The new medicine Fluentem can prevent heart attacks and strokes.”

The first claim manipulated expectedness: here, the claim is unexpected. The second claim was intended to be neutral: we aimed at a prior probability of around 0.5 (representing maximum uncertainty about the truth or falsity of the claim). Participants rated their belief in the second claim.

We predicted that participants would implicitly respond to the expectedness of the first claim, considering it in their view of the proponent's source reliability, and that this source reliability would feed into their assessment of the second claim. Hence, an expected first claim would increase belief in the second claim; an unexpected first claim would decrease it.

In content and design, the materials for Exp. 3 were otherwise based on those of the previous studies. As before, the design was between-subjects: some participants saw expected claims followed by neutral claims (the expected condition), some unexpected followed by neutral claims (the unexpected condition). To aid interpretation of any implicit revision of source reliability in response to expected vs. unexpected claims, we added a third, baseline condition which presented participants only with the neutral, second claim (the null condition).

Given this design, three comparisons are possible: the expected vs. null conditions; the unexpected vs. null conditions; and the expected vs. unexpected conditions. Perceived reliability should increase in response to an expected claim and decrease in response to an unexpected claim. This is in turn should translate into higher ratings of the neutral second claim in the expected than in the unexpected condition. The null condition would be expected to lie between these two, though there are no predictions concerning how far it should be from either, as it reflects how trusting people are initially. Given the differences in design, we anticipated a smaller effect, and therefore increased the sample size.

### Methods

#### Participants

Two samples were recruited on Amazon Mechanical Turk using the same criteria as for Experiments 2a and b; they were again remunerated at a rate of $.20 per minute. Two separate HITs were posted on different days and at different times of day. Participants were able to participate only in one HIT, and only if they had not previously taken part in Experiment 2 a or b. The first sample comprised 120 people (45 women; average age 35.98). The second sample comprised 296 people (3 gender non-conformist, 123 female; average age 34.81). All participants gave informed consent. Note that, although classical methods do not allow samples to be compiled, Bayesian methods do (Kruschke, 2015).

#### Materials and procedure

This experiment used the same set of sources and first claims as Experiments 2a/b. The new, second claim in each pair was as follows: for the valium item, “The new medicine Fluentem can prevent heart attacks and strokes;” for the oven item, “Pimlico Farm superfine flour is the best on the market for making pasta;” for the horse-racing item, “The yacht Azure will beat its competitor Orion at this year's Cowes Week regatta;” for the Stockholm item, “It rained on 13 days in Tübingen, Germany, in May 2013;” for the clubbing item, “Kate Siggs is a rising star on the vibrant Australian jazz scene.” Participants provided a rating for the second claim on a scale from 0 (not at all convincing) to 10 (completely convincing).

To illustrate the new format:

Imagine you hear Robert, who is a senior sports reporter and has predicted the winner in the last 10 races that he covered, telling someone the following: “The Australian horse Thunderbolt, who has beaten the British horse Lightening in the majority of the races entered this season, will lose to (beat) Lightening in the upcoming Cheltenham Festival races.”

Later, Robert tells you the following: “The yacht Azure will beat its competitor Orion at this year's Cowes Week regatta.”

How convincing is this claim about Azure on a scale from 0 (not at all convincing) to 10 (completely convincing)?

The initial claims could be expected or unexpected. In a third condition, there was no initial claim; participants simply rated the neutral claim. Full materials for all conditions are found in the Appendix in Supplementary Material.

### Results

To analyze the data we averaged the endorsement of the second claim across items to create a mean score for each participant. We then ran the analyses on these scores. There are three relevant analyses: expected condition vs. null condition; unexpected condition vs. null condition; expected condition vs. unexpected condition.

As with the one-group analyses, these analyses describe the data with a *t*-distribution, and estimate the most credible parameter values given the data. For the two-group analyses, the following model applies:

$$\begin{array}{l} \mathrm{Pr}\left(\mu_{1},\mu_{2},\sigma_{1},\sigma_{2},\nu |D\right)=\\ \frac{\text{Pr}\left(D|\mu_{1},\mu_{2},\sigma_{1}{,\sigma }_{2},\nu \right)\;\times \text{ Pr}\left(\mu_{1}{,\mu }_{2},\sigma_{1},\sigma_{2},\nu \right)\;}{\text{Pr}\left(D\right)} \end{array}$$

Subscripts identify group membership. Note that, in this model, there is only one parameter for normality. The technical details are the same as for the one-group analyses. The priors are, likewise, set in the same way. Below, for brevity's sake, we report estimates for the differences between *μ*_1_ and *μ*_2_ and between *σ*_1_ and *σ*_2_, for the normality parameter, and for the effect size.

**Figure 10:**
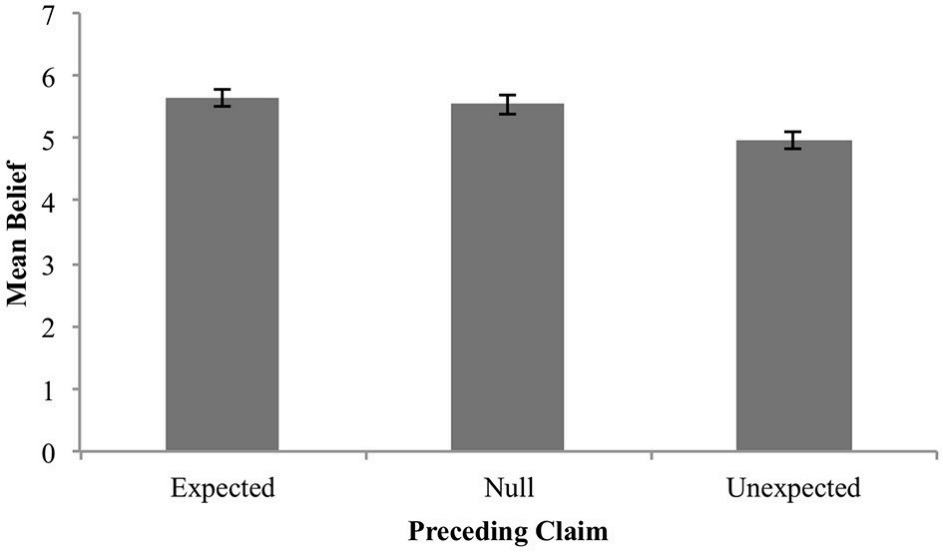
Mean belief in claim by expectedness condition. Error bars are standard error.

Figure 10 shows the descriptive data.

The descriptive data show the predicted pattern in that mean belief was higher in the expected condition, than in the neutral or unexpected condition. The fact that the unexpected condition showed lower mean belief than the null condition is also suggestive of “anti-reliability.”

However, only the contrast between expected and unexpected is statistically reliable, so that no firm conclusions about anti-reliability can be reached in this study.

In detail, the analyses yielded the following.

#### Expected vs. null

**Figure 11:**
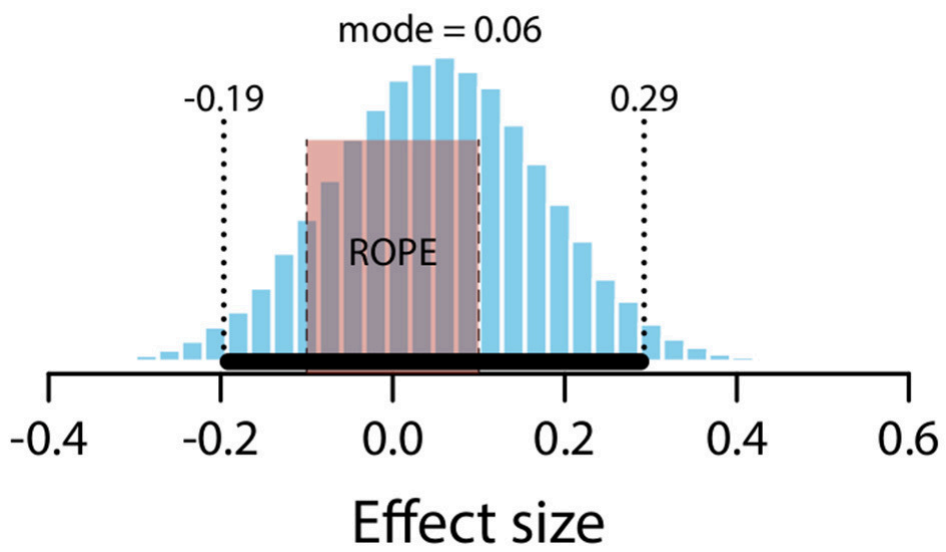
Posterior distribution of effect size for difference between expected and null conditions. ROPE from −0.1 to 0.1. Black bar represents 95% HDI.

The mean estimate for difference in means (*μ*_*expected*_ – *μ*_*null*_) was 0.09 (95% HDI [−0.33, 0.51]). Note that the 95% HDI includes a difference of zero. The modal estimate for difference in standard deviations (*σ*_*expected*_ – *σ*_*null*_) was −0.25 (95% HDI [−0.54, 0.07]). The modal estimate for *ν* was 1.72 (95% HDI [1.22, 2.17]). The modal estimate for effect size was .059 (95% HDI [−0.19, 0.29]). Figure 11 shows the posterior distribution of effect size and the ROPE. Since the 95% HDI for effect size encompasses a conventional ROPE, there is insufficient evidence to determine an effect of preceding expected claims here.

#### Unexpected vs. null

**Figure 12:**
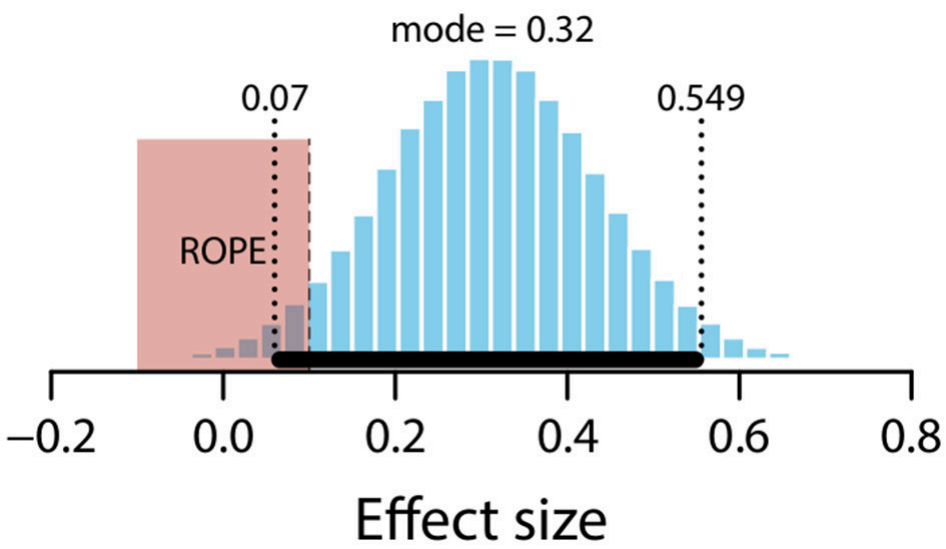
Posterior distribution of effect size for difference between unexpected and null conditions. ROPE from −0.1 to 0.1. Black bar represents 95% HDI.

The mean estimate for difference in means (*μ*_*null*_ – *μ*_*unexpected*_) was 0.56 (95% HDI [0.12, 0.98]), suggesting a credible difference in means, with the unexpected condition lower than the null condition. The modal estimate for difference in standard deviations (*σ*_*null*_ – *σ*_*unexpected*_) was 0.13 (95% HDI [−0.19, 0.44]). The modal estimate for *ν* was 1.73 (95% HDI [1.23, 2.18]). The modal estimate for effect size was 0.32 (95% HDI [0.07, 0.55]). Notice that the 95% HDI excludes an effect size of zero. However, it also overlaps with the conventional ROPE (−0.1 to 0.1) by 0.3. Figure 12 shows the posterior distribution of effect size and the ROPE. Although these data are suggestive, they do not allow us to decide whether or not there is a credible difference between unexpected and null conditions.

#### Expected vs. unexpected

**Figure 13:**
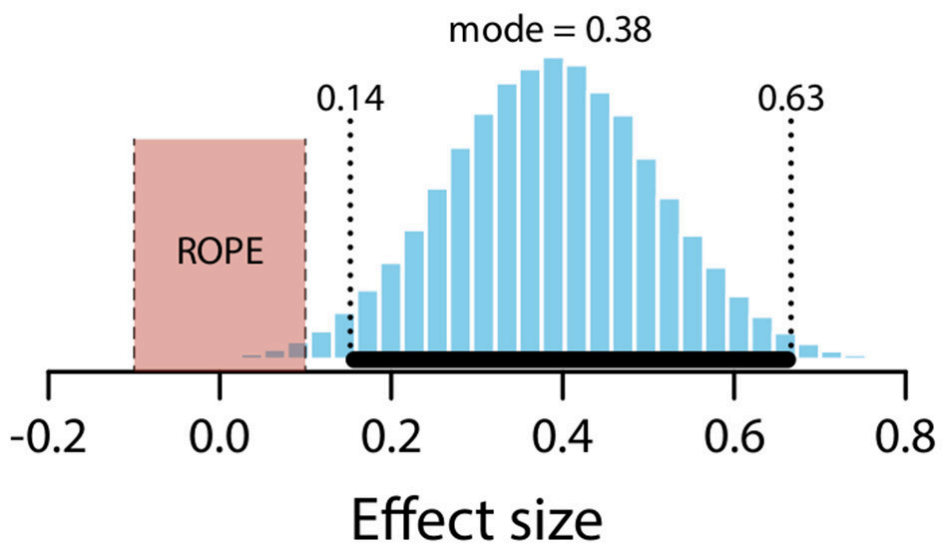
Posterior distribution of effect size for difference between expected and unexpected conditions. ROPE from −0.1 to 0.1. Black bar represents 95% HDI.

The mean estimate for difference in means (*μ*_*expected*_ – *μ*_*unexpected*_) was 0.64 (95% HDI [0.24, 1.05]). The modal estimate for difference in standard deviations (*σ*_*expected*_ – *σ*_*unexpected*_) was −0.1 (95% HDI [−0.4, 0.2]). The modal estimate for *ν* was 1.63 (95% HDI [1.1, 2.13]). The modal estimate for effect size was 0.38 (95% HDI [0.14, 0.63]). The 95% HDI falls outside the conventional ROPE. Figure 13 shows the posterior distribution of the effect size and the ROPE. There is therefore a credible difference between expected and unexpected conditions.

### Discussion

Experiment 3 tested whether participants used perceptions of source reliability generated by a first claim when they assessed a second claim. The expectedness of the first claim affected how participants rated a second claim, indicating that participants implicitly evaluated the first claim in its implications for the source's reliability and updated their beliefs in reliability accordingly. They then brought the resultant beliefs about reliability to bear on their evaluation of a subsequent claim by this source. Claim expectedness thus seems to moderate spontaneous perceptions of reliability even where reliability is not the focus of attention.

## General discussion

Most of the time, arguments, and persuasive messages more generally, come to us from others. As sources, these others will typically be only partially reliable. Understanding how people deal with the perceived reliability of sources is thus an important issue not just for psychology, but also for related disciplines.

The experiments presented tested how people's behavior corresponds to two Bayesian models for handling claims from sources that are only partially reliable. These models posit a Bayesian update process that uses the content of messages or arguments we receive to update both our beliefs about the relevant content (including its implications for other propositions) and our beliefs about the reliability of the reporting source. Our data suggest a reasonable fit between participants' behavior and Olsson and Angere's model of source reliability. Experiments 1a and 2a showed that participants clearly revised their belief upwards given messages from reliable sources and downwards given messages from unreliable sources. This downward revision, or source anti-reliability, is the crucial distinction between the two models: source anti-reliability suggests a closer correspondence with Olsson and Angere's model.

Experiments 1b and 2b broadly supported the predictions of both models concerning belief revision about source reliability. The clearest effect was expected claims improving ratings of source reliability: both experiments supported this result. There was a less clear effect of unexpected claims lowering ratings of source reliability: Experiment 1b showed a statistically credible effect, but the data in Experiment 2b did not allow a decision either way. Experiment 3 tested whether participants behaved similarly when assessing two claims from the same source. In this study, source reliability was entirely implicit. Again a difference was found in belief in a message endorsed by a source, depending on whether that source had previously uttered something expected or unexpected.

In short, when people process messages from others, their processing shows a bi-directional relationship between message content and message source, even in minimal contexts. In the following, we discuss the implications of our findings, both in theoretical and in practical terms.

First, the results seem directly relevant to the practical concern of belief polarization, a phenomenon seen in many real world domains such as politics (see, e.g., Mann and Ornstein, 2012). If people downgrade the perceived reliability of others on the basis of message content this will foster polarization, because opposing views will be discounted (Hahn and Harris, 2014). This process is distinct from having other types of information that might make one downgrade perceived reliability, such as being told that a person has lied or been proven wrong in the past. Specifically, the unexpected messages used in our study were not known by participants to be wrong; they were merely unexpected: in particular, they did not receive ratings of 0 in the initial belief assessment (Experiment 1a: *M* = 2.96; Experiment 2a: *M* = 4.31). In this sense, it is not “track record” of past accuracy that is being monitored but only an *expectation* about accuracy, that is, a “likely track record.” The range of cases where this kind of revision about source reliability would be possible is vastly greater than the number of cases where we have specific information that what someone has just said is wrong. The potential contribution to polarization of the mechanism observed in this paper is consequently considerable. Polarization in the real world will be further accelerated where people are willing to credit others with differing opinions with “anti-reliability,” because now evidence provided by a particular source is taken to constitute actual evidence to the contrary (“ If X says climate change is real, it must be a lie”). Again, this seems a likely contributing factor in political views and debate.

On a theoretical level, the results match intuitions in present Bayesian models of testimonial evidence (Bovens and Hartmann, 2003; Olsson and Vallinder, 2013). This carries through to the models of argument that have incorporated aspects of the BH model, such as those experimentally tested in Harris et al. (2012) and Harris et al. (2015). Of course, alternative Bayesian accounts differ in detail (such as the difference with respect to anti-reliability we considered here). Which account is preferable from a normative perspective requires further consideration. However, descriptively, we found at least some evidence of anti-reliability. At the same time these and other Bayesian accounts (including for example, Schum, 1981; Shafto et al., 2012; Fenton et al., 2013) all share the basic property that source and content factors will never have just a simple additive relationship. This fundamental feature stems from the multiplicative nature of Bayes' rule as the “engine” of Bayesian models.

Data from past experimental studies are compatible with such multiplicative relationships. Particularly relevant are studies which examine the relationship between argument content and argument source from a Bayesian perspective (e.g., Hahn et al., 2009; Harris et al., 2013). Such studies have found statistical interactions between argument content and argument source. Manipulating argument quality and source reliability gives rise to non-additive effects on perceived argument strength: high quality arguments from reliable sources are *more* convincing than an additive model would predict.

This multiplicative relationship conflicts with ELM postulates for a number of reasons. First, the materials of those studies involve hypothetical scenarios that should be considered low personal involvement and hence low elaboration. Hahn et al. (2009) study, for example, involves a (fictitious) high energy sports drink “FIZZ” whose qualities are recommended either through a message giving facts and figures or reporting (anonymous) personal opinions—in line with argument quality considerations put forward in the ELM literature (Petty et al., 1981, p. 850). For the source manipulation, these arguments are put forward either in a “circular email from excitingnews@wowee.com” in the low reliability condition or, in the high reliability condition a “report by an independent consumer watchdog.” It thus seems unlikely that the observed interactions between argument content and source could be generated because the source manipulation gave rise to the materials being processed in very different ways. Yet these argumentation results are clearly relevant to the ELM: what is studied in the context of “rational argument” within cognitive psychology is not an alternative to persuasion; it is a *subset* of persuasion—namely that subset that rests on argument quality within the domain of “analytic processing.”

In the present paper, Experiments 1 and 2, again motivated by a Bayesian perspective, demonstrate a further type of interaction between message source and message content, namely the observed bi-directional relationship. This relationship, too, conflicts with the ELM. Again, the materials are “low involvement.” This in itself makes it somewhat surprising that they show effects predicted by models that are aimed at analytic processing. However, it is also not clear how a dual-route model that, by default, factors source and content considerations into alternative routes can naturally deal with our results. Of course, it is easy enough from an ELM perspective to explain the effects of source reliability on message convincingness through heuristic processing that treats source reliability as a simple cue. However, it is more difficult to explain the reverse direction: how message convincingness affects source reliability. Why should the mere plausibility of message content have an effect on source reliability, and why should that feed back into the evaluation of further content?

Experiment 3 emphasizes further this difficulty for the ELM. Differences in perceived source reliability give rise to differential effects on message convincingness, yet the relevant difference in source reliability is only brought about by differences in (prior) message content. In other words, message content must be processed to infer source reliability, which is then brought to bear on the subsequent simple message. This is further evidence of a bi-directional relationship between content and source: a relationship that surfaces in materials that the ELM would assign to a single (peripheral, heuristic) route. The ELM, then, seems to drive too large a wedge between source and content considerations. From a Bayesian perspective, source and content should be tightly coupled, and our data suggest that they are indeed much more richly inter-twined than the standard model of persuasion has assumed.

Much remains to be done with respect to a full understanding of the relationship between message content and source reliability information in argumentation and persuasion. What exactly are the boundary conditions for their interaction? Again, we think consideration of normative models and evidential value will be useful here. Certainly there are cases in which source information is obviously relevant: for instance, when an argument from authority or an *ad hominem* argument is being made. However, there are also cases in which source information is not obviously relevant: say, for instance, a deductively valid argument or an inductive argument for which the recipient can check all the relevant facts. Much real world argument, however, lies in between the two extremes of having to rely entirely on another's assessment and being fully able to appreciate an issue oneself.

At the same time, closer scrutiny of normative issues reveals two distinct aspects of source reliability that bear on persuasion. We will call the first aspect the *testimonial aspect*: by this we mean the evidential value of endorsement. The source, in effect, *is* the evidence. Authority X says that Y is the case, and the mere fact that Authority X says this is the evidence that affects one's belief in Y. The evidential value of endorsement by a source will vary with the reliability of the source. On the plausible assumption that, as an authority on the topic, X is more likely to be right than wrong, X's testimony will carry greater evidential weight than will that of a non-expert. The second aspect of source reliability, we will call the *transmission aspect*. Here the source transmits evidence, and source reliability considerations influence our beliefs about the faithfulness of that transmission. For example, when a doctor determines her beliefs about whether a patient has a particular disease, she may base her assessment on the outcome of a medical test. Typically, however, she will not have conducted that test herself, but merely receives a report of the outcome of such a test. Normatively, her confidence in the diagnosis should be affected by the reliability of the reporting source, not just the reported test result itself; in other words, her confidence in the diagnosis should be higher where the test result has been communicated by a reliable lab than by one that is known to have on occasion mixed up patient materials.

Different strands in prior research have focused on different aspects of source reliability. The ELM seems to have been primarily concerned with the testimonial aspect, touching on transmission only where source considerations are assumed to affect processing: for example, where source expertise affects the direction of thoughts (Chaiken and Maheswaran, 1994). The Bayesian argumentation studies involving factorial manipulations of argument content and argument source described earlier (e.g., Hahn et al., 2009) have been concerned with transmission. The present studies focus on the testimonial aspect. However, in everyday life, most examples of persuasive communication will likely involve aspects of both.

Consequently, a better understanding of the role of source characteristics in argumentation and persuasion will need to be aware of, and examine in more detail, these different facets.

## Ethics statement

A preliminary analysis of Experiments 1a and b appeared in the Proceedings of the 37th Annual Meeting of the Cognitive Society. The studies were conducted in line with the ethical guidelines at Lund University and the ethics committee of the Department of Psychological Sciences, Birkbeck, University of London.

## Author contributions

UH, YvG, and EO: designed Experiments 1a and b; YG: collected the data for Experiments 1a and b; All authors contributed to the design of the remaining experiments; PC and UH: collected the data for the remaining experiments, analyzed the data for all experiments, and prepared the original draft; All authors edited and approved the final draft.

### Conflict of interest statement

The authors declare that the research was conducted in the absence of any commercial or financial relationships that could be construed as a potential conflict of interest.

## Footnotes

^1^This exceptionality is also apparent in studies which investigate debiasing from source characteristics (e.g., Petty et al., 1998).

^2^Bayesian belief networks are graphical models that simplify Bayesian computations by characterizing independence relations between variables. Nodes represent variables; links represent dependencies between variables, typically causal connections. For an introduction see e.g., (Korb and Nicholson, 2010).

^3^As will be clear from the Appendix in Supplementary Material, the items differ in how they implemented expectedness. In some cases, expectedness was determined by presumed background knowledge; in other cases, by information present in the items themselves. The cause of the expectedness does not matter from the point of view of probability theory or the models. Nevertheless, it is plausible that the different causes give rise to different psychological processing. We leave exploration of this issue to future work.

^4^The original design aimed to test a somewhat more complex set of predictions than discussed here; hence the 2 × 2 design. However, these predictions required lower scores on prior ratings than were actually achieved. The one-way predictions are appropriate to the achieved values. Descriptives for the full design are contained in Appendix 2 in Supplementary Material.
