# Supplementary material for: The Bi-directional Relationship between Source Characteristics and Message Content
Source: Front Psychol. 2018 Jan 30;9:18. doi: 10.3389/fpsyg.2018.00018 (PMC5797680; doi:10.3389/fpsyg.2018.00018)
Supplement: Supplementary file 1 [file DataSheet1.docx]

# Appendix 1: Materials

## Experiment 1a

Unreliable and reliable sources; unreliable sources in brackets

1. Claim: One of the best remedies against severe cough is valium (lots to drink, hot or cold).

[Convincingness Rating]

Now imagine that Michael, who is a clinical nurse specialist (drug addict), told you the following: ‘One of the best remedies against severe cough is valium (lots to drink, hot or cold).’

[Rating]

2. Claim: The temperature in the Kitchen Mate oven varies a lot (keeps very steady), which is perfect for a fluffy and crispy bread.

[Rating]

Now imagine that Paula, who is a baker (a designer kitchen Kitchen Mate salesperson working on commission), told you the following: ‘The temperature in the Kitchen Mate oven varies a lot (keeps very steady), which is perfect for a fluffy and crispy bread.’

[Rating

3. Claim: The Australian horse ‘Thunderbolt’, who has beaten the British horse ‘Lightening’ in the majority of races entered this season, will lose to (beat) ‘Lightening’ at the upcoming Cheltenham Festival.

[Rating]

Now imagine that Robert, who is a senior sports reporter and has predicted the winner in the last 10 races he covered (a junior sports reporter and has failed to predict…), told you: ‘The Australian horse ‘Thunderbolt’, who has beaten the British horse ‘Lightening’ in the majority of races entered this season, will lose to (beat) ‘Lightening’ at the upcoming Cheltenham Festival.’

4. Claim: In 2013, the maximum temperature in Stockholm in June was 15 degrees (23 degrees)

[Rating]

Now imagine that Mary, who is a retired meteorologist (who is five years old and was given a weather station for Christmas), told you: ‘In 2013, the maximum temperature in Stockholm in June was 15 degrees (23 degrees).’

[Rating]

5. Claim: The Land Rover Discovery has no problems (has problems) with the electricity and very

cheap spare parts.

[Rating]

Now imagine that Patrick, who is a car enthusiast (a used car salesperson), told you: ‘The Land Rover

Discovery has no problems (has problems) with the electricity and very cheap spare parts.’

[Rating]

6. Claim: The Eclipse nightclub in Detroit (Ibiza) has the reputation of one of the coolest nightclubs in the world.

[Rating]

Now imagine that Emma, who is a 26-year-old DJ and has established herself, has a leading figure in the club scene (who is a 45 year old housewife with three young children (between 5 and 12) who enjoys knitting) and regularly attends the Eclipse nightclub with her friends, told you: ‘The Eclipse nightclub in Detroit (Ibiza) has the reputation of one of the coolest nightclubs in the world.’

[Rating]

## Experiment 1b

Source and claim manipulations as above. Texts took the following structure.

Michael is a clinical nurse specialist (drug addict).

[Reliability Rating]

Now imagine that Michael, who is a clinical nurse specialist (drug addict), told you the following: ‘One of the best remedies against severe cough is valium (lots to drink, hot or cold).’

[Reliability Rating]

## Experiment 2a & 2b

Materials as above, except for the following changes. Text 4 was adapted so that temperatures were given in Fahrenheit as well as Centigrade. Text 5 is removed. Text 6 is adapted so that the unexpected claim is that the nightclub is in Des Moines, Iowa, and the expected claim is that the nightclub is in Manhattan.

## Experiment 3

### Expected Claims

1. Imagine you hear Michael, who is a clinical nurse specialist (drug addict), telling someone “One of the best remedies for a severe cough is lots to drink, hot or cold.”

Later, Michael tells you the following: “The new medicine Fluentem can prevent heart attacks and strokes.”

How convincing is this claim about Fluentem on a scale from 0 (not at all convincing) to 10 (completely convincing)?

2. Imagine you hear Paula, who is a baker (a designer kitchen Kitchen Mate salesperson working on commission), telling someone “The temperature in the Kitchen Mate remains constant, which is perfect for a fluffy and crispy bread.”

Later, Paula tells you the following: “Pimlico Farm superfine flour is the best on the market for making pasta.”

How convincing is this claim about Pimlico Farm flour on a scale from 0 (not at all convincing) to 10 (completely convincing)?

3. Imagine you hear Robert, who is a senior sports reporter and has predicted the winner in the last 10 races that he covered (a junior sports reporter and has failed to predict…), telling someone the following: “The Australian horse Thunderbolt, who has beaten the British horse Lightening in the majority of the races entered this season, will beat Lightening in the upcoming Cheltenham Festival races.”

Later, Robert tells you the following: “The yacht Azure will beat its competitor Orion at this year’s Cowes Week regatta.”

How convincing is this claim about Azure on a scale from 0 (not at all convincing) to 10 (completely convincing)?

4.Imagine you hear Mary, who is a retired meteorologist (5 years old and was bought a weather station for Christmas), telling someone “In 2013, the maximum temperature in Stockholm in June was 23 degrees Centigrade/73 degrees Fahrenheit.”

Later, Mary tells you the following: “It rained on 13 days in Tübingen in May 2015.”

How convincing is this claim about Tübingen on a scale from 0 (not at all convincing) to 10 (completely convincing)?

5. Imagine you hear Emma, who is a 26-year-old DJ and has established herself as a leading figure in the club scene (who is a 45 year old housewife with three young children (between 5 and 12) who enjoys knitting) and regularly attends the nightclub with her friends, telling someone the following: “A nightclub in Manhattan has the reputation of one of the coolest nightclubs in the world.”

Later, Emma tells you the following: “Kate Siggs is a rising star on the vibrant Australian jazz scene.”

### Unexpected Claims

1. Imagine you hear Michael, who is a clinical nurse specialist (drug addict), telling someone “One of the best remedies for a severe cough is valium.”

Later, Michael tells you the following: “The new medicine Fluentem can prevent heart attacks and strokes.”

How convincing is this claim about Fluentem on a scale from 0 (not at all convincing) to 10 (completely convincing)?

2. Imagine you hear Paula, who is a baker (a designer kitchen Kitchen Mate salesperson working on commission), telling someone “The temperature in the Kitchen Mate oven varies a lot, which is perfect for a fluffy and crispy bread.”

Later, Paula tells you the following: “Pimlico Farm superfine flour is the best on the market for making pasta.”

How convincing is this claim about Pimlico Farm flour on a scale from 0 (not at all convincing) to 10 (completely convincing)?

3. Imagine you hear Robert, who is a senior sports reporter and has predicted the winner in the last 10 races that he covered (a junior sports reporter and has failed to predict…), telling someone the following: “The Australian horse Thunderbolt, who has beaten the British horse Lightening in the majority of the races entered this season, will lose to Lightening in the upcoming Cheltenham Festival races.”

Later, Robert tells you the following: “The yacht Azure will beat its competitor Orion at this year’s Cowes Week regatta.”

How convincing is this claim about Azure on a scale from 0 (not at all convincing) to 10 (completely convincing)?

4.Imagine you hear Mary, who is a retired meteorologist (5 years old and was bought a weather station for Christmas), telling someone “In 2013, the maximum temperature in Stockholm in June was 15 degrees Centigrade/59 degrees Fahrenheit.”

Later, Mary tells you the following: “It rained on 13 days in Tübingen in May 2015.”

How convincing is this claim about Tübingen on a scale from 0 (not at all convincing) to 10 (completely convincing)?

5. Imagine you hear Emma, is a 26-year-old DJ and has established herself as a leading figure in the club scene (who is a 45 year old housewife with three young children (between 5 and 12) who enjoys knitting), telling someone the following: “A nightclub in Des Moines, Iowa, has the reputation of one of the coolest nightclubs in the world.”

Later, Emma tells you the following: “Kate Siggs is a rising star on the vibrant Australian jazz scene.”

How convincing is this claim about Kate Siggs on a scale from 0 (not at all convincing) to 10 (completely convincing)?

### Null Condition

1. Imagine that Michael, who is a clinical nurse specialist (drug addict), tells you the following: “The new medicine Fluentem can prevent heart attacks and strokes.”

How convincing is this claim about Fluentem on a scale from 0 (not at all convincing) to 10 (completely convincing)?

2. Imagine that, Paula, who is a baker (a designer kitchen Kitchen Mate salesperson working on commission), tells you the following: “Pimlico Farm superfine flour is the best on the market for making pasta.”

How convincing is this claim about Pimlico Farm flour on a scale from 0 (not at all convincing) to 10 (completely convincing)?

3. Imagine that Robert, who is a senior sports reporter and has predicted the winner in the last 10 races that he covered (a junior sports reporter and has failed to predict…), tells you the following: “The yacht Azure will beat its competitor Orion at this year’s Cowes Week regatta.”

How convincing is this claim about Azure on a scale from 0 (not at all convincing) to 10 (completely convincing)?

4. Imagine that Mary, who is a retired meteorologist (5 years old and was bought a weather station for Christmas), tells you the following: “It rained on 13 days in Tübingen in May 2015.”

How convincing is this claim about Tübingen on a scale from 0 (not at all convincing) to 10 (completely convincing)?

5. Imagine that Emma ,who is a 26-year-old DJ and has established herself as a leading figure in the club scene (who is a 45 year old housewife with three young children (between 5 and 12) who enjoys knitting), tells you the following: “Kate Siggs is a rising star on the vibrant Australian jazz scene.”

How convincing is this claim about Kate Siggs on a scale from 0 (not at all convincing) to 10 (completely convincing)?

# Appendix 2: Supplementary statistics

Table 12.1. Prior convincingness as a manipulation check. These figures are the result of Bayesian independent-samples t-tests: the mean of the posterior distribution of difference of meanings and the 95% HDI.

|  |  | Parameter Estimates |  |
| --- | --- | --- | --- |
| Experiment | Comparison | Mean Posterior Difference of Means | 95% HDI |
| Experiment 1a | Expected > Unexpected Claim | 2.25 | [1.5, 3] |
| Experiment 1b | Reliable > Unreliable Sources | 2.08 | [1.63, 2.55] |
| Experiment 2a | Expected > Unexpected Claim | 1.69 | [1.04, 2.35] |
| Experiment 2b | Reliable > Unreliable Sources | 2.8 | [2.36, 3.24] |

Table 12.2. Descriptive statistics for full design of Experiment 1a. The figures are mean belief change, standard deviation in brackets

|  | Expected | Unexpected |
| --- | --- | --- |
| Reliable | 1.71 (1.58) | 2.09 (1.38) |
| Unreliable | -.78 (1.87) | -.67 (1.27) |

Table 12.3. Descriptive statistics for full design of Experiment 1b. The figures are mean change in reliability, standard deviation in brackets

|  | Expected | Unexpected |
| --- | --- | --- |
| Reliable | .56 (.92) | -1.43 (1.50) |
| Unreliable | .35 (1.01) | -.49 (1.16) |

Table 12.4. Descriptive statistics for full design of Experiment 2a. The figures are mean belief change, standard deviation in brackets

|  | Expected | Unexpected |
| --- | --- | --- |
| Reliable | 1.72(1.36) | 2.73(1.35) |
| Unreliable | -1.86(1.53) | -1.61(1.80) |

Table 12.5. Descriptives for full design of Experiment 2b. The figures are mean change in reliability, standard deviation in brackets

|  | Expected | Unexpected |
| --- | --- | --- |
| Reliable | .54 (1.01) | -.99 (1.70) |
| Unreliable | .45 (.96) | .31 (.86) |
